# *PAX5* fusion genes in t(7;9)(q11.2;p13) leukemia: a case report and review of the literature

**DOI:** 10.1186/1755-8166-7-13

**Published:** 2014-02-07

**Authors:** Dagmar Denk, Jutta Bradtke, Margit König, Sabine Strehl

**Affiliations:** 1 CCRI, Children’s Cancer Research Institute, St. Anna Kinderkrebsforschung e.V., Zimmermannplatz 10, 1090 Vienna, Austria; 2 Universitätsklinikum Gießen und Marburg, Institut für Pathologie, Onkogenetisches Labor Molekularpathologie, Langhansstrasse 10, 35392 Gießen, Germany

**Keywords:** B-cell precursor acute lymphoblastic leukemia, t(7;9)(q11.2;p13), der(9)t(7;9)(q11.2;p13), *PAX5*-fusions, *ELN*, *AUTS2*, *POM121*

## Abstract

**Background:**

B-cell precursor acute lymphoblastic leukemia (BCP-ALL) is characterized by recurrent genetic alterations including chromosomal translocations. The transcription factor *PAX5*, which is pivotal for B-cell commitment and maintenance, is affected by rearrangements, which lead to the expression of in-frame fusion genes in about 2.5% of the cases.

**Results:**

Using conventional cytogenetics, fluorescence in situ hybridization (FISH), and molecular methods, an additional case with a der(9)t(7;9)(q11.23;p13) resulting in the expression of a *PAX5-ELN* fusion gene was identified. Furthermore, a general review of leukemia harboring a t(7;9)(q11.2;p13) or der(9)t(7;9)(q11.2;p13), which occurs more often in children than in adults and shows a remarkably high male preponderance, is given. These cytogenetically highly similar translocations lead to the expression of one of three different in frame *PAX5*-fusions, namely with *AUTS2* (7q11.22), *ELN* (7q11.23), or *POM121* (7q11.23), which constitute the only currently known cluster of *PAX5* partner genes.

**Conclusion:**

Our report underlines the recurrent involvement of *PAX5* in different fusion genes resulting either from t(7;9)(q11.2;p13) or der(9)t(7;9)(q11.2;p13), which cannot be distinguished cytogenetically and whose discrimination requires molecular analysis.

## Background

*PAX5* rearrangements, resulting in the expression of in-frame fusion genes, account for about 2.5% of pediatric B-cell precursor acute lymphoblastic leukemia (BCP-ALL) [1]. While several groups, including our own, have reported the incidence and diversity of *PAX5* fusion genes [1-7], their occurrence in leukemia harboring a t(7;9)(q11.2;p13) or der(9)t(7;9)(q11.2;p13) has not yet been investigated in detail. Herein, we describe an additional case with a *PAX5-ELN* fusion and summarize the demographic and genetic data of all cases with t(7;9)(q11.2;p13)/der(9)t(7;9)(q11.2;p13) leukemia reported to date.

## Case presentation

**Figure 1:**
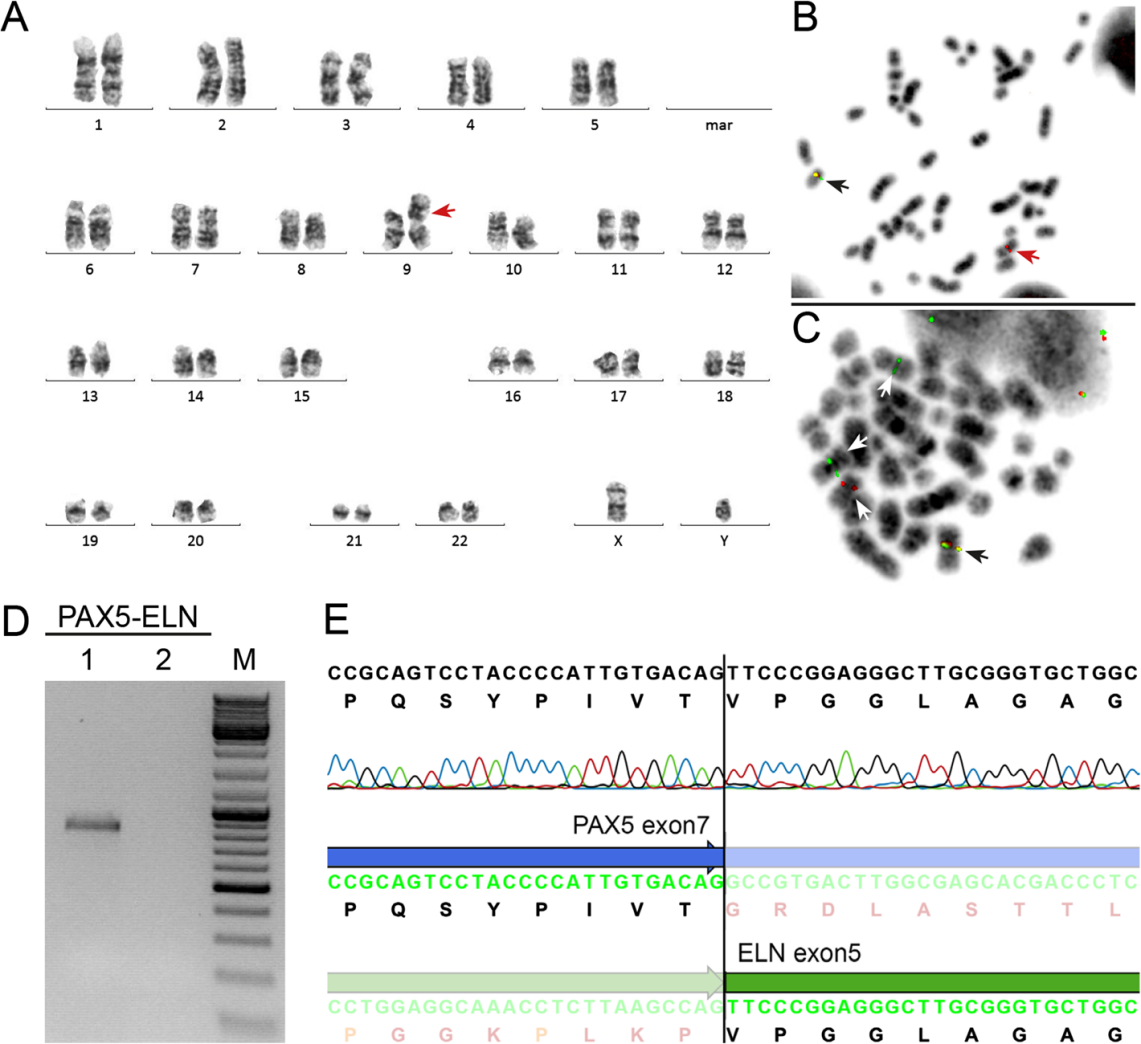
**Cytogenetic and molecular genetic analysis of a *PAX5-ELN *positive case. (A)** Karyogram; red arrow indicates the derivative chromosome der(9)t(7;9)(q11.23;p13) (refined karyotype using molecular methods). **(B)** FISH using *PAX5*-specific BAC clones showing a 3′-end deletion: 5′-end-specific clone (red signals); 3′-end-specific clone (green signals); black and red arrows indicate the normal and derivative chromosome, respectively. **(C)** FISH using *PAX5-* and *ELN*-specific BAC clones showing a co-localization: *PAX5* 5′-end-specific clone (red signals); *ELN* 3′-end-specific clone (green signals); arrows indicate the normal chromosomes 9 and 12 (white) and the derivative chromosome (black). **(D)** RT-PCR using primers located in *PAX5* exon 2–3 and *ELN* exon 6 resulting in amplification of *PAX5-ELN* fusion transcripts. M, molecular weight marker DNA-mix ladder (Peqlab); lane 1, patient No. 5; lane 2, normal control. **(E)** Sequence chromatogram of the *PAX5-ELN* fusion junction showing the fusion between exon 7 of *PAX5* and exon 5 of *ELN*.

**Figure 2:**
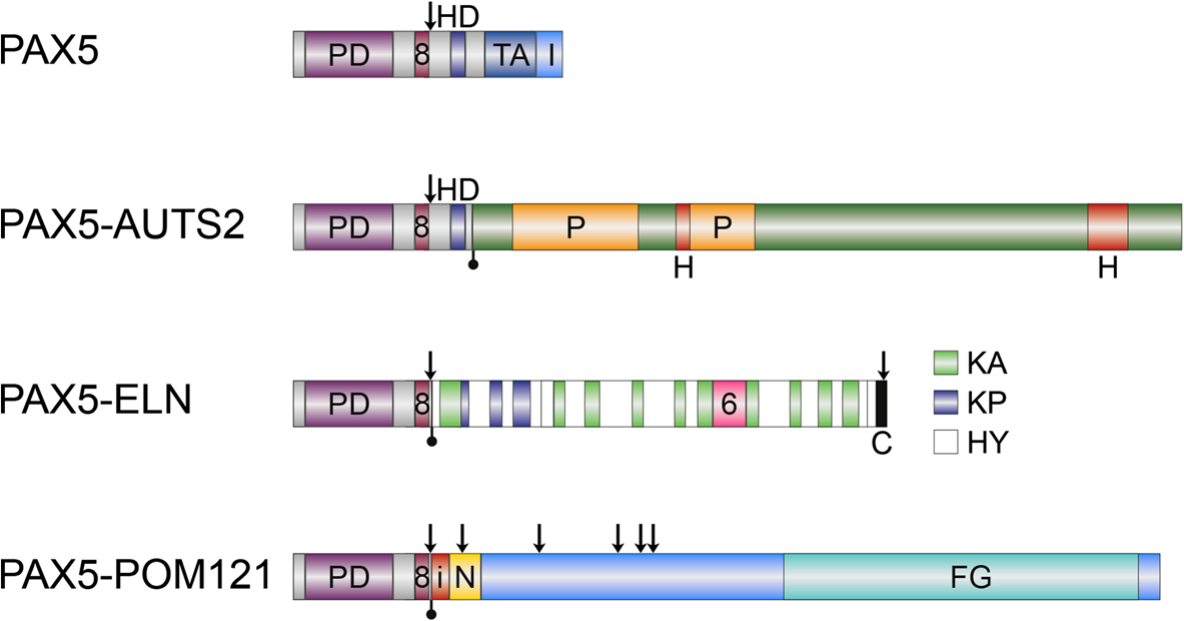
**Schematic representation of the structure of PAX5 and the putative consensus chimeric proteins.** PD, paired domain; 8, octapeptide; HD, partial homeodomain; TA, transactivation domain; I, inhibitory domain; P, proline-rich regions; H, histidine-rich regions; KA, alanine-rich cross-linking domains; KP, proline-rich cross-linking domains; HY, hydrophobic domains; 6, VGVAPG hexapeptide domain; C, C-terminal domain; i, insertion; N, POM121 5′-untranslated region; FG, FG-repeats; arrows and filled lollipops indicate nuclear localization signals and fusion breakpoints, respectively.

We have identified an additional case of pediatric BCP-ALL with an infrequent der(9)t(7;9)(q11.23;p13) resulting in the expression of an in-frame *PAX5-ELN* fusion gene (Table 1). Cytogenetic analysis of the bone marrow of a 19.4-year-old adolescent revealed - together with many secondary aberrations - a der(9)t(7;9)(q11.2;p13) (Figure 1A) and subsequent FISH analysis using *PAX5*-flanking BAC clones showed a deletion of the 3′-end-specific probe, which is suggestive of a *PAX5* gene rearrangement (Figure 1B). Further FISH analysis, using *PAX5*- and *ELN*-specific clones, identified *ELN* as the fusion partner (Figure 1C), which was verified on the molecular level by RT-PCR (Figure 1D). Sequencing of the amplification product showed that exon 7 of *PAX5* was fused to exon 5 of *ELN* (Figure 1E). In all *PAX5-ELN* cases, except for one in which *PAX5* exon 5 was fused to *ELN* sequences, the breakpoints in *PAX5* occurred in intron 7 [2,4,8]. Also, the breakpoints in *ELN* are heterogeneous and *PAX5* is fused to either exon 2 or exon 5 of *ELN* (Table 1) [2,4,8]. Consequently, the consensus PAX5-ELN fusion protein consists of the DNA-binding paired domain, the octapeptide, and the nuclear localization signal of PAX5, which are fused to almost the entire ELN protein without the signal peptide (Figure 2).

**Table 1:** Demographic and genetic data of t(7;9)(q11.2;p13) and der(9)t(7;9)(q11.2;p13) positive B-ALL cases

**Case**	**Age**	**Sex**	**Phenotype**	**Karyotype**	***PAX5 *** **fusion gene**	**Reference**
1	37	M	B-III	46,XY,t(7;9)(q11;p13)[17]	PAX5 ex7/ELN ex2	[2]
2	16	M	B-II	46,XY,t(7;9)(q11;p13),del(9)(p21)[14]/	PAX5 ex7/ELN ex2	[2]
				46,XY[1]		
3	4	M	B-I	46,XY,t(7;9)(q11;p13)[2]/	PAX5 ex7/ELN ex2	[4]
				46,XY[18]		
4	1.4	M	B-ALL	NA (9906_037)	PAX5 ex5/ELN*	[8]
5	19.4	M	B-III	46,XY,?add(4)(q2?),?add(5)(p14),del(8)(p21),der(9)t(7;9)(q11.2;p13),	PAX5 ex7/ELN ex5	This work
				del(16)(p13),inc[cp16]/46,idem,?dup(1)(q21)[3]/46,XY[4]		
6	3.1	M	B-III	45,XY,-7,der(9)t(7;9)(q11;p13)[15]/	PAX5 ex6/AUTS2 ex4	[5]
				46,XY[1]		
7	2.8	F	B-III	45,XX,-7,der(9)t(7;9)(q11;p13),dup(16)(p11p13)[14]/	PAX5 ex6/AUTS2 ex6	[9]
				46,XX[2]		
8	0.6	M	B-II/III	46,XY,t(7;9)(q11;p13)[8]	PAX5 ex6/AUTS2 ex5	[10]
9	2.1	M	B-III	46,XY,del(7)(q22q33)?,del(9)(q22?),del(12)(p11)[8]	PAX5 ex5/POM121 ex4	[1]
10	1	M	B-III	46,XY,t(7;9)(q11;p13)[21]/	PAX5 ex5/POM121 ex4	[4]
				46,XY[3]		
11	1.7	F	B-ALL	45,XX,-7,der(9)t(7;9)(q11;p13)[17]	NA	[12]
				46,XX[2]		
12	1.3	M	B-ALL	45,XY,inv(1)(p35q32),-7,der(9)t(7;9)(q11;p13)[31]	NA	[13]
				46,XY[17]		

All cases, except cases 4 and 9, are listed in the Mitelman database from which also the respective karyotypes have been retrieved [11].

*PAX5 exon 5 is fused to an unknown *ELN* exon.

NA, data not available.

## Results and discussion

So far, sixteen in-frame *PAX5*-fusions have been described and the fusion partners comprise a heterogeneous group of genes encoding proteins, which play distinct roles in signaling, transcription, chromatin remodeling, and cell structuring [1-7,9,10]. Three of the sixteen currently known *PAX5* fusion partners, namely, *AUTS2*, *ELN*, and *POM121*, are located at the pericentromeric region of 7q and encompass roughly 3.3 Mb of genomic DNA, forming the only currently known cluster of *PAX5* partner genes. Therefore, t(7;9)(q11.2;p13) translocations may give rise to three different recurrent fusion genes, i.e. *PAX5-AUTS2*, *PAX5-POM121*, and *PAX5-ELN* (Figure 2), which are not distinguishable at the cytogenetic level. In addition, karyotyping of five of the cases showed an unbalanced der(9)t(7;9)(q11.2;p13) with loss of the reciprocal derivative chromosome (Table 1; cases 5–7 and 11–12). One of the cases was identified by SNP array (case 4), only detecting unbalanced chromosome alterations; furthermore, most cases showed a deletion of the *PAX5* 3′-end by FISH, further supporting the notion that the *PAX5-*partner fusions, and not the reciprocal ones contribute to leukemogenesis.

t(7;9)(q11.2;p13)/der(9)t(7;9)(q11.2;p13) positive leukemia is a rare disease and only 9 cases have been collected in the Mitelman database of the cancer genome anatomy project ([11] accessed November 2013) (Table 1). Three of these cases were *PAX5-ELN* positive [2,4] and in addition, a case with a *PAX5-ELN* fusion without cytogenetic data has been reported [8]. Together, including the case described herein, five patients harboring this fusion gene have now been identified.

Other cases involving the cluster of *PAX5* fusion partners include: Three patients with a *PAX5-AUTS2*[5,9,10], two with a *PAX5-POM121* fusion gene [1,4], and in two cases involvement of *PAX5* has not been investigated [12,13] (Table 1). Of note, in the *PAX5-POM121* case we have previously published [1], cytogenetic analysis failed to identify a t(7;9)(q11.2;p13), but the chromosome quality was rather poor. Whole chromosome painting with probes specific for chromosomes 7 and 9 showed the presence of a der(7;9), on which the 3′-end of *PAX5* was located, whereas the 5′-end of *PAX5*, generating the *PAX5-POM121* fusion, was translocated to a derivative chromosome, which only partially consisted of chromosome 7 material (data not shown). Together, with the molecular data that showed an insertion of chromosome 12 sequences in the fusion, a more complex rearrangement with involvement of at least chromosomes 7, 9, and 12 generated the in-frame *PAX5-POM121* fusion [1].

Furthermore, out of the 12 cases with t(7;9)(q11.2;p13)/der(9)t(7;9)(q11.2;p13) rearrangements only one was an adult and two were young adolescents, whereas all other patients were ≤ 4 years of age (Table 1), suggesting that this subtype of leukemia occurs more frequently in pediatric than in adult cases. Remarkably, 83% (10/12) of the t(7;9)(q11.2;p13)/der(9)t(7;9)(q11.2;p13) patients were male, and thus, the male/female ratio was 5. Although the number of so far reported cases is rather low, in acute leukemia such an extreme gender bias is exceedingly rare [14]. This finding is intriguing, but currently there is no plausible explanation why a specific subtype of leukemia is associated with one or the other gender.

Regarding the prognostic relevance of *PAX5* fusion genes in general, due to their rareness no final conclusions may be drawn. However, we have recently shown that *PAX5-AUTS2* leukemia may have a rather unfavorable outcome [10]. Out of the five *PAX5-ELN* cases, one patient (case 4) showed high-risk features and displayed a *JAK1* mutation and a *BCR-ABL1*-like expression signature [8]. Furthermore, cases 1 and 2 both relapsed post allograft and died 16 months after initial diagnosis ([15] accessed November 2013). The *PAX5-ELN* positive patient presented herein is currently, eight months after initial diagnosis, in complete remission. Together, there is at least some evidence that t(7;9)(q11.2;p13)/der(9)t(7;9)(q11.2;p13) leukemia may have a rather poor prognosis. However, whether this is attributable to the specific *PAX5*-fusions or to coinciding mutations in, for example, tyrosine kinases, remains to be determined, and a larger cohort of patients needs to be analyzed, which, due to the low incidence of this leukemia subtype, will require an international collaborative effort.

## Conclusion

In this report an additional case of *PAX5-ELN* positive leukemia is described, and, furthermore, an overview of the published cases of t(7;9)(q11.2;p13)/der(9)t(7;9)(q11.2;p13) leukemia is given, emphasizing the importance of molecular analysis to discriminate between cytogenetically identical translocations resulting in distinct fusion genes.

## Material and methods

### Cytogenetic and fluorescence in situ hybridization (FISH) analysis

Cytogenetic analysis was performed according to standard techniques. FISH analysis using *PAX5*- and *ELN*-specific probes was conducted as previously described [1]. The *PAX5* rearrangement was first detected using *PAX5*-flanking BAC clones RP11-220I1 and RP11-12P15 (obtained from Pieter de Jong, BACPAC Resources, Children’s Hospital and Research Center Oakland, CA, USA). Verification of the *PAX5-ELN* fusion was performed using the *PAX5* 5′-end-flanking BAC clone RP11-220I1 in combination with the *ELN* 3′-end-specific clone RP11-349P21 (Welcome Trust Sanger Institute; http://www.sanger.ac.uk).

### RNA isolation and reverse transcription-polymerase chain reaction (RT-PCR)

RNA isolation and RT-PCR for the detection of *PAX5*-*ELN* transcripts were performed according to standard procedures using primers PAX5ex2-3-F1 (5′-TCTTGGCAGGTATTAT GAGACAGGAAG-3′) and ELNex6-R2 (5′-AGCAGCGTCAGCCACTCCAC-3′) located in exons 2–3 and 6 of *PAX5* and *ELN*, respectively. Amplification products were directly sequenced (Microsynth AG, Austria) and sequence analysis was conducted using the CLC Main Workbench 6.0 (CLC bio, Denmark).

### Reference sequences and exon nomenclature

The chromosome band positions of the genes and the exon nomenclature used correspond to that of the Ensemble database and the reference sequences for *AUTS2* (ENST00000342771), *ELN* (ENST00000252034), *POM121* (ENST00000257622), and *PAX5* (ENST00000358127) (Ensembl release 73 - September 2013). A summary of all mRNA fusion sequences as well as the entire transcript and protein sequences of the putative consensus chimeras PAX5-AUTS2, PAX5-ELN, and PAX5-POM121 are provided as Additional file 1.

## Consent

Within the AIEOP-BFM ALL 2009 study (ClinicalTrials.gov Identifier: NCT01117441), written informed consent - which includes the compliance that surplus material not required for diagnostic purposes may be used for research purposes - is obtained from the patients, their parents or their legal guardians. This study has exclusively been performed on material obtained for diagnostic purposes and neither any additional medical intervention nor patient recruitment was necessary.

## Competing interests

The authors declare that they have no competing interest.

## Authors’ contributions

DD conducted experiments, analyzed the data, and wrote the manuscript; MK conducted FISH analysis; JB performed cytogenetic analysis; SS supervised the study and drafted the manuscript. All authors read and approved the final manuscript.

## Supplementary Material

Additional file 1 PAX5 fusion genes in t(7;9)(q11;p13) leukemia: A case report and review of the literature. Click here for file
