# Supplementary material for: PAX5 fusion genes in t(7;9)(q11.2;p13) leukemia: a case report and review of the literature
Source: Mol Cytogenet. 2014 Feb 7;7:13. doi: 10.1186/1755-8166-7-13 (PMC3937052; doi:10.1186/1755-8166-7-13)
Supplement: Additional file 1 — PAX5 fusion genes in t(7;9)(q11;p13) leukemia: A case report and review of the literature. [file 1755-8166-7-13-S1.docx]

**Additional file 1: PAX5 fusion genes in t(7;9)(q11;p13) leukemia: A case report and review of the literature**

A summary of all mRNA fusion sequences as well as the entire transcript and protein sequences of the putative consensus chimeras PAX5-AUTS2, PAX5-ELN, and PAX5-POM121.

**PAX5-AUTS2**

PAX5ex6-AUTS2ex4 [[1](#_ENREF_1)]

...CATCAAGCCCGAGCAGAGTTCAGCTCC...

PAX5ex6-AUTS2ex6 [[2](#_ENREF_2)]

...ATCAAGCCCGAGCAGGCATCAGATGCCAGC...

PAX5ex6-AUTS2ex5 [[3](#_ENREF_3)]

...TCCGGGCAGAGACTTCCTCCGGAAGCAGATGCGGGGAGACTTGTTCACACAGCAGCAGCTGGAGGTGCTGGACCGCGTGTTTGAGAGGCAGCACTACTCAGACATCTTCACCACCACAGAGCCCATCAAGCCCGAGCAGTGTGACAGTGACAGTGACCAGGAAGAGAAGGCATCAGATGCCAGCTCTGAAAAACTCTTCAACACTGTTATTGTAAACAAAGATCCGGAGTTAGGTGTTGGCACGCTACCAGAACATGACAGCCAGGATGCAGGGCCGATTGTCCCCAAGATATCGGGTCTAGAGAGAAGCCAGGAGAAGAGCCAGGACTGTTGCAAAGAGCCAATCTTTGAGCCTGTGGTGCTTAAAGACCCCTGCCCTCA...

**PAX5-ELN**

PAX5ex7-ELNex2 [[4](#_ENREF_4)]

…TACCCCATTGTGACAGGGGTCCCTGGGGCCATT…

PAX5ex5-ELN [[5](#_ENREF_5)]

no sequence available

PAX5-ELN [[6](#_ENREF_6)]

no sequence available

PAX5ex7-ELNex5 (this work)

…TGACATCGGGAGCAGTGTGCCAGGCCCGCAGTCCTACCCCATTGTGACAGTTCCCGGAGGGCTTGCGGGTGCTGGCCTTGGGGCAGGGCTCGGCGCCTTCCCCGCAGT…

**PAX5-POM121**

PAX5ex5-POM121ex4 [[7](#_ENREF_7)]

...CACTGGCTCCGTGACGCAGGTGTCCTCGGTGAGCACGGATTCGGCCGGCTCGTCGTACTCCATCAGCGGCATCCTGGGCATCACGTCCCCCAGCGCCGACACCAACAAGCGCAAGAGAGACGAAGGACAGAGGGCAAACACTGCCATCACATCTATGAAGCAGGAAACAGCCCTCACTGGACACCAAATCTGCCAGCCTCTTGATCTTGGACTTCCTAGCCTCCGGAACTGTGAAAAGGATTGTGGGACTTTACCAAATCGGTTTGTAATAACACCTAGAAGACGCTATCCGATCCATCAGGCCCAGTATTCCTGTCTGGGGGTACTTCCCACCGTGTGCTGGAATGGTTATCACAAGAAGGCTGTGCTGTCCCCT...

(intersticial coding sequence)

PAX5ex5-POM121ex4 [[6](#_ENREF_6)]

…GTGTCCTCGGTGAGCACGGATTCGGCCGGCTCGTCGTACTCCATCAGCGGCATCCTGGGCATCACGTCCCCCAGCGCCGACACCAACAAGCGCAAGAGAGACGAAGATCACCTGAAAGTATCTTGGGGACCCCTTCTTCCAACCCTGGCAGGGCTCCCTGGACTACACTTTGAAAATTTCTGCTTACCAAATCGGTTTGTAATAACACCTAGAAGACGCTATCCGATCCATCAGGCCCAGTATTCCTGTCTGGGGGTACTTCCCACCGTGTGCTGGAATGGTTATCACAAGAAGGCTGTGCTGTCCCCTCGCAACTCCAGGATGGTGTGTAGCCCAGTGACTGTGAGGAT…

(first 13bp of exon4 missing)

(intersticial coding sequence)

**>consensus transcript PAX5-AUTS2 (3870bp)**

ATGGATTTAGAGAAAAATTATCCGACTCCTCGGACCAGCAGGACAGGACATGGAGGAGTGAATCAGCTTGGGGGGGTTTTTGTGAATGGACGGCCACTCCCGGATGTAGTCCGCCAGAGGATAGTGGAACTTGCTCATCAAGGTGTCAGGCCCTGCGACATCTCCAGGCAGCTTCGGGTCAGCCATGGTTGTGTCAGCAAAATTCTTGGCAGGTATTATGAGACAGGAAGCATCAAGCCTGGGGTAATTGGAGGATCCAAACCAAAGGTCGCCACACCCAAAGTGGTGGAAAAAATCGCTGAATATAAACGCCAAAATCCCACCATGTTTGCCTGGGAGATCAGGGACCGGCTGCTGGCAGAGCGGGTGTGTGACAATGACACCGTGCCTAGCGTCAGTTCCATCAACAGGATCATCCGGACAAAAGTACAGCAGCCACCCAACCAACCAGTCCCAGCTTCCAGTCACAGCATAGTGTCCACTGGCTCCGTGACGCAGGTGTCCTCGGTGAGCACGGATTCGGCCGGCTCGTCGTACTCCATCAGCGGCATCCTGGGCATCACGTCCCCCAGCGCCGACACCAACAAGCGCAAGAGAGACGAAGGTATTCAGGAGTCTCCGGTGCCGAACGGCCACTCGCTTCCGGGCAGAGACTTCCTCCGGAAGCAGATGCGGGGAGACTTGTTCACACAGCAGCAGCTGGAGGTGCTGGACCGCGTGTTTGAGAGGCAGCACTACTCAGACATCTTCACCACCACAGAGCCCATCAAGCCCGAGCAGGCATCAGATGCCAGCTCTGAAAAACTCTTCAACACTGTTATTGTAAACAAAGATCCGGAGTTAGGTGTTGGCACGCTACCAGAACATGACAGCCAGGATGCAGGGCCGATTGTCCCCAAGATATCGGGTCTAGAGAGAAGCCAGGAGAAGAGCCAGGACTGTTGCAAAGAGCCAATCTTTGAGCCTGTGGTGCTTAAAGACCCCTGCCCTCAGGTCGCACAGCCAATACCCCAGCCGCAGACGGAGCCCCAACTCCGAGCTCCTTCTCCGGACCCTGACTTGGTGCAGCGCACAGAGGCCCCACCTCAACCCCCACCTCTGAGTACACAGCCACCACAGGGCCCTCCTGAGGCCCAGCTCCAGCCTGCCCCGCAGCCTCAGGTGCAGAGGCCACCCAGGCCACAGTCCCCCACCCAGCTGCTCCATCAGAACCTCCCACCTGTGCAGGCCCACCCCTCTGCTCAGAGCCTCTCCCAGCCATTGTCAGCCTACAACAGCAGTAGCTTAAGCCTCAACAGTTTAAGCAGCAGCAGAAGCAGCACTCCAGCGAAGACTCAGCCCGCCCCACCTCACATCTCCCACCACCCCTCTGCCTCCCCGTTCCCCCTCTCCCTGCCCAACCACAGCCCCCTGCACAGCTTCACACCCACCCTCCAGCCCCCCGCACACTCACATCACCCCAATATGTTTGCCCCTCCCACTGCTCTGCCTCCTCCACCACCACTGACATCAGGAAGTCTGCAGGTGGCCGGACACCCGGCCGGGAGCACTTACTCAGAGCAAGACATCTTGCGACAGGAACTGAACACTCGTTTTTTGGCCTCTCAGAGTGCTGACCGCGGGGCTTCCCTGGGCCCTCCGCCCTACCTGCGGACCGAGTTCCATCAGCACCAGCACCAGCACCAGCACACCCACCAGCACACGCACCAGCACACCTTCACGCCGTTCCCCCACGCCATCCCACCCACCGCCATCATGCCGACGCCAGCACCTCCCATGTTTGACAAATACCCTACAAAAGTTGACCCATTCTACCGGCACAGTCTCTTCCATTCCTATCCTCCTGCAGTGTCGGGCATCCCCCCTATGATCCCACCCACTGGCCCTTTTGGTTCACTACAAGGAGCATTTCAGCCGAAGACATCCAACCCTATCGATGTCGCTGCTCGGCCTGGGACAGTCCCACACACTTTACTCCAAAAGGACCCGAGGTTGACAGATCCTTTCAGACCTATGTTAAGGAAACCAGGGAAGTGGTGTGCTATGCATGTTCACATCGCCTGGCAGATTTACCACCACCAACAGAAAGTCAAGAAACAGATGCAGTCAGACCCACATAAGCTGGACTTTGGACTGAAACCTGAGTTCCTGAGCCGCCCTCCAGGCCCCAGTCTTTTTGGAGCCATCCACCACCCCCATGACCTGGCACGGCCTTCAACTTTGTTCTCTGCCGCTGGTGCTGCACACCCAACTGGGACCCCTTTTGGGCCACCTCCTCATCACAGCAACTTCCTCAACCCTGCTGCCCACCTAGAGCCTTTTAATCGGCCGTCTACATTCACAGGCCTAGCAGCAGTTGGTGGCAATGCCTTCGGGGGACTTGGAAATCCTTCCGTTACACCCAACTCAATGTTCGGCCACAAGGATGGCCCCAGTGTGCAGAACTTTAGCAACCCTCACGAACCCTGGAACCGGCTGCACCGAACGCCTCCGTCGTTCCCGACCCCTCCGCCCTGGCTGAAGCCAGGGGAGCTGGAGCGCAGCGCGTCCGCTGCAGCTCATGACAGAGATAGAGATGTAGATAAACGAGACTCATCTGTTAGTAAAGATGACAAAGAAAGGGAAAGCGTCGAGAAGAGACACTCCAGCCACCCTTCACCAGCACCTGTCCTCCCGGTGAATGCCCTGGGACATACCCGCAGCTCCACTGAACAGATCCGGGCTCATCTGAACACTGAGGCTCGGGAGAAGGACAAACCCAAAGAGAGGGAGAGAGACCACTCGGAATCCCGCAAGGACCTGGCCGCCGACGAGCACAAGGCGAAAGAGGGCCACCTGCCCGAGAAGGACGGGCACGGCCACGAGGGGCGCGCCGCGGGCGAAGAGGCCAAGCAGCTGGCCCGGGTGCCGTCTCCCTACGTGCGGACCCCGGTGGTGGAGAGTGCCAGGCCCAACAGCACCTCGAGCCGGGAGGCCGAGCCGCGCAAGGGTGAGCCGGCCTACGAGAACCCCAAGAAGAGCTCCGAGGTCAAGGTGAAGGAGGAGCGGAAGGAAGACCATGACCTGCCTCCAGAGGCCCCGCAGACCCACCGGGCCTCGGAGCCGCCGCCTCCCAACTCCTCGTCCAGCGTGCACCCGGGGCCCCTGGCCTCGATGCCCATGACGGTGGGGGTGACGGGCATTCACCCCATGAACAGCATCAGCAGCCTGGACAGGACTCGCATGATGACCCCCTTCATGGGCATCAGCCCCCTCCCGGGCGGAGAGCGCTTCCCGTACCCTTCTTTCCACTGGGACCCCATCCGGGACCCCTTGAGGGATCCTTACCGAGAACTTGACATTCACCGGAGAGACCCGCTGGGCAGGGACTTCCTGCTAAGGAACGACCCGCTCCACCGGCTCTCGACTCCCCGGCTGTACGAAGCCGACCGCTCCTTCAGGGACCGGGAGCCTCACGACTACAGCCACCACCACCACCACCACCACCACCCGCTGTCTGTGGACCCTCGGCGGGAGCACGAGCGGGGAGGCCACCTGGACGAGCGGGAGCGCTTGCACATGCTCAGAGAAGACTACGAGCACACGCGGCTCCACTCCGTGCACCCCGCCTCCCTCGACGGACACCTCCCCCACCCCAGCCTCATCACCCCGGGACTCCCCAGCATGCACTATCCCCGCATCAGCCCCACCGCGGGCAACCAGAACGGACTCCTCAACAAGACCCCTCCGACAGCAGCGCTGAGCGCACCTCCCCCGCTCATCTCCACGCTGGGGGGCCGCCCGGTCTCTCCCAGAAGGACGACTCCTCTGTCCGCAGAGATAAGGGAGAGGCCCCCTTCCCACACGCTGAAGGATATCGAGGCCCGATAA

**>consensus protein PAX5-AUTS2 (1290 AA)**

MDLEKNYPTPRTSRTGHGGVNQLGGVFVNGRPLPDVVRQRIVELAHQGVRPCDISRQLRVSHGCVSKILGRYYETGSIKPGVIGGSKPKVATPKVVEKIAEYKRQNPTMFAWEIRDRLLAERVCDNDTVPSVSSINRIIRTKVQQPPNQPVPASSHSIVSTGSVTQVSSVSTDSAGSSYSISGILGITSPSADTNKRKRDEGIQESPVPNGHSLPGRDFLRKQMRGDLFTQQQLEVLDRVFERQHYSDIFTTTEPIKPEQASDASSEKLFNTVIVNKDPELGVGTLPEHDSQDAGPIVPKISGLERSQEKSQDCCKEPIFEPVVLKDPCPQVAQPIPQPQTEPQLRAPSPDPDLVQRTEAPPQPPPLSTQPPQGPPEAQLQPAPQPQVQRPPRPQSPTQLLHQNLPPVQAHPSAQSLSQPLSAYNSSSLSLNSLSSSRSSTPAKTQPAPPHISHHPSASPFPLSLPNHSPLHSFTPTLQPPAHSHHPNMFAPPTALPPPPPLTSGSLQVAGHPAGSTYSEQDILRQELNTRFLASQSADRGASLGPPPYLRTEFHQHQHQHQHTHQHTHQHTFTPFPHAIPPTAIMPTPAPPMFDKYPTKVDPFYRHSLFHSYPPAVSGIPPMIPPTGPFGSLQGAFQPKTSNPIDVAARPGTVPHTLLQKDPRLTDPFRPMLRKPGKWCAMHVHIAWQIYHHQQKVKKQMQSDPHKLDFGLKPEFLSRPPGPSLFGAIHHPHDLARPSTLFSAAGAAHPTGTPFGPPPHHSNFLNPAAHLEPFNRPSTFTGLAAVGGNAFGGLGNPSVTPNSMFGHKDGPSVQNFSNPHEPWNRLHRTPPSFPTPPPWLKPGELERSASAAAHDRDRDVDKRDSSVSKDDKERESVEKRHSSHPSPAPVLPVNALGHTRSSTEQIRAHLNTEAREKDKPKERERDHSESRKDLAADEHKAKEGHLPEKDGHGHEGRAAGEEAKQLARVPSPYVRTPVVESARPNSTSSREAEPRKGEPAYENPKKSSEVKVKEERKEDHDLPPEAPQTHRASEPPPPNSSSSVHPGPLASMPMTVGVTGIHPMNSISSLDRTRMMTPFMGISPLPGGERFPYPSFHWDPIRDPLRDPYRELDIHRRDPLGRDFLLRNDPLHRLSTPRLYEADRSFRDREPHDYSHHHHHHHHPLSVDPRREHERGGHLDERERLHMLREDYEHTRLHSVHPASLDGHLPHPSLITPGLPSMHYPRISPTAGNQNGLLNKTPPTAALSAPPPLISTLGGRPVSPRRTTPLSAEIRERPPSHTLKDIEAR-

**>consensus transcript PAX5-ELN (2889bp)**

ATGGATTTAGAGAAAAATTATCCGACTCCTCGGACCAGCAGGACAGGACATGGAGGAGTGAATCAGCTTGGGGGGGTTTTTGTGAATGGACGGCCACTCCCGGATGTAGTCCGCCAGAGGATAGTGGAACTTGCTCATCAAGGTGTCAGGCCCTGCGACATCTCCAGGCAGCTTCGGGTCAGCCATGGTTGTGTCAGCAAAATTCTTGGCAGGTATTATGAGACAGGAAGCATCAAGCCTGGGGTAATTGGAGGATCCAAACCAAAGGTCGCCACACCCAAAGTGGTGGAAAAAATCGCTGAATATAAACGCCAAAATCCCACCATGTTTGCCTGGGAGATCAGGGACCGGCTGCTGGCAGAGCGGGTGTGTGACAATGACACCGTGCCTAGCGTCAGTTCCATCAACAGGATCATCCGGACAAAAGTACAGCAGCCACCCAACCAACCAGTCCCAGCTTCCAGTCACAGCATAGTGTCCACTGGCTCCGTGACGCAGGTGTCCTCGGTGAGCACGGATTCGGCCGGCTCGTCGTACTCCATCAGCGGCATCCTGGGCATCACGTCCCCCAGCGCCGACACCAACAAGCGCAAGAGAGACGAAGTTCCCGGAGGGCTTGCGGGTGCTGGCCTTGGGGCAGGGCTCGGCGCCTTCCCCGCAGTTACCTTTCCGGGGGCTCTGGTGCCTGGTGGAGTGGCTGACGCTGCTGCAGCCTATAAAGCTGCTAAGGCTGGCGCTGGGCTTGGTGGTGTCCCAGGAGTTGGTGGCTTAGGAGTGTCTGCAGGTGCGGTGGTTCCTCAGCCTGGAGCCGGAGTGAAGCCTGGGAAAGTGCCGGGTGTGGGGCTGCCAGGTGTATACCCAGGTGGCGTGCTCCCAGGAGCTCGGTTCCCCGGTGTGGGGGTGCTCCCTGGAGTTCCCACTGGAGCAGGAGTTAAGCCCAAGGCTCCAGGTGTAGGTGGAGCTTTTGCTGGAATCCCAGGAGTTGGACCCTTTGGGGGACCGCAACCTGGAGTCCCACTGGGGTATCCCATCAAGGCCCCCAAGCTGCCTGGTGGCTATGGACTGCCCTACACCACAGGGAAACTGCCCTATGGCTATGGGCCCGGAGGAGTGGCTGGTGCAGCGGGCAAGGCTGGTTACCCAACAGGGACAGGGGTTGGCCCCCAGGCAGCAGCAGCAGCGGCAGCTAAAGCAGCAGCAAAGTTCGGTGCTGGAGCAGCCGGAGTCCTCCCTGGTGTTGGAGGGGCTGGTGTTCCTGGCGTGCCTGGGGCAATTCCTGGAATTGGAGGCATCGCAGGCGTTGGGACTCCAGCTGCAGCTGCAGCTGCAGCAGCAGCCGCTAAGGCAGCCAAGTATGGAGCTGCTGCAGGCTTAGTGCCTGGTGGGCCAGGCTTTGGCCCGGGAGTAGTTGGTGTCCCAGGAGCTGGCGTTCCAGGTGTTGGTGTCCCAGGAGCTGGGATTCCAGTTGTCCCAGGTGCTGGGATCCCAGGTGCTGCGGTTCCAGGGGTTGTGTCACCAGAAGCAGCTGCTAAGGCAGCTGCAAAGGCAGCCAAATACGGGGCCAGGCCCGGAGTCGGAGTTGGAGGCATTCCTACTTACGGGGTTGGAGCTGGGGGCTTTCCCGGCTTTGGTGTCGGAGTCGGAGGTATCCCTGGAGTCGCAGGTGTCCCTGGTGTCGGAGGTGTTCCCGGAGTCGGAGGTGTCCCGGGAGTTGGCATTTCCCCCGAAGCTCAGGCAGCAGCTGCCGCCAAGGCTGCCAAGTACGGAGTGGGGACCCCAGCAGCTGCAGCTGCTAAAGCAGCCGCCAAAGCCGCCCAGTTTGGGTTAGTTCCTGGTGTCGGCGTGGCTCCTGGAGTTGGCGTGGCTCCTGGTGTCGGTGTGGCTCCTGGAGTTGGCTTGGCTCCTGGAGTTGGCGTGGCTCCTGGAGTTGGTGTGGCTCCTGGCGTTGGCGTGGCTCCCGGCATTGGCCCTGGTGGAGTTGCAGCTGCAGCAAAATCCGCTGCCAAGGTGGCTGCCAAAGCCCAGCTCCGAGCTGCAGCTGGGCTTGGTGCTGGCATCCCTGGACTTGGAGTTGGTGTCGGCGTCCCTGGACTTGGAGTTGGTGCTGGTGTTCCTGGACTTGGAGTTGGTGCTGGTGTTCCTGGCTTCGGGGCAGTACCTGGAGCCCTGGCTGCCGCTAAAGCAGCCAAATATGGAGCAGCAGTGCCTGGGGTCCTTGGAGGGCTCGGGGCTCTCGGTGGAGTAGGCATCCCAGGCGGTGTGGTGGGAGCCGGACCCGCCGCCGCCGCTGCCGCAGCCAAAGCTGCTGCCAAAGCCGCCCAGTTTGGCCTAGTGGGAGCCGCTGGGCTCGGAGGACTCGGAGTCGGAGGGCTTGGAGTTCCAGGTGTTGGGGGCCTTGGAGGTATACCTCCAGCTGCAGCCGCTAAAGCAGCTAAATACGGTGCTGCTGGCCTTGGAGGTGTCCTAGGGGGTGCCGGGCAGTTCCCACTTGGAGGAGTGGCAGCAAGACCTGGCTTCGGATTGTCTCCCATTTTCCCAGGTGGGGCCTGCCTGGGGAAAGCTTGTGGCCGGAAGAGAAAATGA

**>consensus protein PAX5-ELN (963 AA)**

MDLEKNYPTPRTSRTGHGGVNQLGGVFVNGRPLPDVVRQRIVELAHQGVRPCDISRQLRVSHGCVSKILGRYYETGSIKPGVIGGSKPKVATPKVVEKIAEYKRQNPTMFAWEIRDRLLAERVCDNDTVPSVSSINRIIRTKVQQPPNQPVPASSHSIVSTGSVTQVSSVSTDSAGSSYSISGILGITSPSADTNKRKRDEVPGGLAGAGLGAGLGAFPAVTFPGALVPGGVADAAAAYKAAKAGAGLGGVPGVGGLGVSAGAVVPQPGAGVKPGKVPGVGLPGVYPGGVLPGARFPGVGVLPGVPTGAGVKPKAPGVGGAFAGIPGVGPFGGPQPGVPLGYPIKAPKLPGGYGLPYTTGKLPYGYGPGGVAGAAGKAGYPTGTGVGPQAAAAAAAKAAAKFGAGAAGVLPGVGGAGVPGVPGAIPGIGGIAGVGTPAAAAAAAAAAKAAKYGAAAGLVPGGPGFGPGVVGVPGAGVPGVGVPGAGIPVVPGAGIPGAAVPGVVSPEAAAKAAAKAAKYGARPGVGVGGIPTYGVGAGGFPGFGVGVGGIPGVAGVPGVGGVPGVGGVPGVGISPEAQAAAAAKAAKYGVGTPAAAAAKAAAKAAQFGLVPGVGVAPGVGVAPGVGVAPGVGLAPGVGVAPGVGVAPGVGVAPGIGPGGVAAAAKSAAKVAAKAQLRAAAGLGAGIPGLGVGVGVPGLGVGAGVPGLGVGAGVPGFGAVPGALAAAKAAKYGAAVPGVLGGLGALGGVGIPGGVVGAGPAAAAAAAKAAAKAAQFGLVGAAGLGGLGVGGLGVPGVGGLGGIPPAAAAKAAKYGAAGLGGVLGGAGQFPLGGVAARPGFGLSPIFPGGACLGKACGRKRK-

**>consensus transcript PAX5-insert-POM121 (3774bp, 1258 AA)**

ATGGATTTAGAGAAAAATTATCCGACTCCTCGGACCAGCAGGACAGGACATGGAGGAGTGAATCAGCTTGGGGGGGTTTTTGTGAATGGACGGCCACTCCCGGATGTAGTCCGCCAGAGGATAGTGGAACTTGCTCATCAAGGTGTCAGGCCCTGCGACATCTCCAGGCAGCTTCGGGTCAGCCATGGTTGTGTCAGCAAAATTCTTGGCAGGTATTATGAGACAGGAAGCATCAAGCCTGGGGTAATTGGAGGATCCAAACCAAAGGTCGCCACACCCAAAGTGGTGGAAAAAATCGCTGAATATAAACGCCAAAATCCCACCATGTTTGCCTGGGAGATCAGGGACCGGCTGCTGGCAGAGCGGGTGTGTGACAATGACACCGTGCCTAGCGTCAGTTCCATCAACAGGATCATCCGGACAAAAGTACAGCAGCCACCCAACCAACCAGTCCCAGCTTCCAGTCACAGCATAGTGTCCACTGGCTCCGTGACGCAGGTGTCCTCGGTGAGCACGGATTCGGCCGGCTCGTCGTACTCCATCAGCGGCATCCTGGGCATCACGTCCCCCAGCGCCGACACCAACAAGCGCAAGAGAGACGAAGATCACCTGAAAGTATCTTGGGGACCCCTTCTTCCAACCCTGGCAGGGCTCCCTGGACTACACTTTGAAAATTTCTGCTTACCAAATCGGTTTGTAATAACACCTAGAAGACGCTATCCGATCCATCAGGCCCAGTATTCCTGTCTGGGGGTACTTCCCACCGTGTGCTGGAATGGTTATCACAAGAAGGCTGTGCTGTCCCCTCGCAACTCCAGGATGGTGTGTAGCCCAGTGACTGTGAGGATCGCCCCTCCTGACAGAAGATTTTCGCGTTCTGCGATACCAGAGCAGATAATCAGCTCAACACTGTCCTCACCATCAAGTAACGCCCCAGACCCATGTGCAAAGGAGACAGTACTGAGTGCCCTCAAAGAGAAGGAGAAGAAAAGGACAGTGGAGGAAGAAGACCAAATATTCCTTGATGGCCAGGAAAATAAAAGAAGGCGCCATGATAGCAGTGGCAGTGGACATTCAGCATTTGAGCCCCTGGTGGCCAATGGAGTCCCCGCTTCTTTTGTGCCTAAGCCTGGGTCTCTGAAGAGAGGCCTCAATTCTCAGAGCTCAGATGACCACTTGAATAAGAGATCCCGAAGCTCTTCCATGAGCTCCTTGACAGGCGCTTACGCAAGTGGCATCCCTAGCTCCAGCCGCAATGCCATTACCAGTTCCTACAGCTCCACTCGAGGCATCTCACAGCTCTGGAAGAGAAATGGCCCCAGTTCATCACCCTTCTCTAGCCCAGCCTCCTCCCGCTCCCAGACACCGGAGAGGCCAGCAAAGAAAATAAGAGAAGAGGAGCTGTGTCATCATTCCAGTTCTTCAACTCCATTGGCAGCAGACAGGGAGTCCCAGGGAGAAAAGGCTGCAGATACAACCCCAAGGAAGAAACAAAACTCGAATTCTCAGTCTACACCTGGCAGCTCTGGGCAGCGTAAGCGGAAAGTTCAGCTGCTGCCTTCTCGGCGAGGGGAACAGCTGACCTTGCCTCCACCTCCCCAGCTTGGCTATTCGATCACTGCCGAGGACCTAGACTTAGAGAAGAAGGCTTCATTACAGTGGTTCAACCAGGCCTTGGAGGACAAGAGCGATGCTGCCTCGAACTCTGTCACTGAGACCCCACCTATCACTCAGCCTTCATTTACCTTTACCCTGCCTGCTGCTGCACCTGCCTCCCCACCCACCTCCCTCCTGGCCCCAAGCACCAACCCACTGTTAGAGAGCTTGAAGAAGATGCAGACTCCCCCGAGCCTGCCACCCTGCCCAGAATCTGCTGGAGCAGCAACCACTGAGGCCCTCTCACCTCCAAAGACACCCAGCCTCCTACCCCCGCTGGGTTTATCACAGTCAGGGCCGCCAGGGCTGCTCCCCAGCCCCTCCTTTGACTCCAAACCCCCGACCACTTTGCTGGGGCTGATCCCTGCTCCATCCATGGTACCAGCCACTGACACCAAGGCACCTCCAACCCTTCAGGCAGAGACGGCTACCAAACCCCAAGCCACATCTGCCCCGTCCCCCGCCCCCAAGCAAAGCTTCCTGTTTGGAACACAGAACACCTCACCTTCCAGCCCTGCCGCCCCTGCTGCATCTTCAGCACCTCCCATGTTCAAGCCCATTTTCACGGCTCCACCCAAGAGTGAGAAGGAAGGCCCCACACCGCCTGGCCCTTCAGTCACAGCCACAGCGCCCTCCAGCTCCTCCCTCCCCACGACCACCAGCACCACAGCCCCGACCTTCCAGCCTGTCTTTAGCAGCATGGGGCCACCTGCATCTGTGCCCTTGCCTGCTCCCTTCTTCAAGCAGACAACTACTCCCGCCACTGCTCCCACCACAACTGCCCCGCTCTTCACTGGCCTGGCCAGCGCCACCTCTGCTGTGGCTCCCATCACCTCTGCCAGTCCATCCACAGACTCTGCTTCGAAGCCTGCGTTTGGCTTTGGCATAAACAGTGTGAGCAGCAGCAGTGTGAGTACCACGACCAGCACCGCCACTGCCGCCTCACAGCCTTTCCTCTTCGGGGCGCCCCAGGCCTCTGCTGCCAGCTTCACCCCGGCCATGGGCTCCATATTCCAGTTTGGCAAACCTCCTGCCTTGCCCACAACCACCACAGTCACCACCTTCAGCCAGTCCCTGCACACTGCCGTGCCAACGGCCACCAGCAGCAGCGCTGCCGACTTTAGTGGTTTTGGCAGCACCCTCGCCACCTCCGCCCCGGCCACCAGCAGCCAGCCCACTCTGACGTTCAGTAACACGAGCACCCCCACGTTCAACATTCCCTTTGGCTCAAGCGCCAAGTCCCCGCTCCCATCATATCCGGGAGCCAACCCCCAGCCCGCATTTGGGGCCGCTGAGGGGCAGCCACCGGGGGCCGCCAAGCCGGCCCTTGCCCCCAGCTTTGGCAGCTCTTTCACTTTTGGAAACTCTGCAGCCCCGGCTGCTGCACCCACACCTGCACCTCCGTCCATGATCAAGGTCGTGCCTGCGTACGTGCCTACGCCCATCCATCCTATCTTTGGCGGTGCCACGCACTCGGCGTTTGGGTTGAAAGCCACGGCTTCGGCCTTCGGCGCTCCCGCCAGCTCACAGCCCGCCTTTGGCGGCTCCACTGCTGTCTTCTTCGGTGCAGCCACCAGCTCCGGCTTTGGAGCCACCACCCAGACCGCCAGCAGCGGGAGCAGCAGCTCGGTGTTTGGCAGCACAACACCATCACCCTTCACGTTTGGGGGTTCGGCAGCCCCCGCTGGCAGTGGGAGCTTTGGGATCAATGTGGCCACCCCAGGCTCCAGCACCACCACCGGAGCTTTCAGCTTTGGAGCAGGACAGAGTGGGAGCACAGCCACCTCCACCCCCTTCGCAGGGGGCTTAGGTCAGAACGCCCTGGGCACCACCGGCCAGAGCACACCGTTTGCCTTCAACGTGAGCAGCACAACTGAGAGCAAACCTGTGTTTGGAGGCACCGCCACCCCCACCTTTGGTCTGAACACCCCTGCGCCTGGAGTGGGCACATCAGGCAGCAGCCTCTCCTTTGGGGCATCCTCAGCACCCGCCCAAGGCTTTGTTGGTGTTGCACCTTTCGGATCGGCGGCCCTTTCATTTTCCATTGGTGCGGGATCCAAGACCCCAGGGGCTCGACAGCGACTGCAGGCCCGAAGGCAGCACACCCGCAAAAAGTAG

**>consensus protein PAX5-POM121 (1258 AA)**

MDLEKNYPTPRTSRTGHGGVNQLGGVFVNGRPLPDVVRQRIVELAHQGVRPCDISRQLRVSHGCVSKILGRYYETGSIKPGVIGGSKPKVATPKVVEKIAEYKRQNPTMFAWEIRDRLLAERVCDNDTVPSVSSINRIIRTKVQQPPNQPVPASSHSIVSTGSVTQVSSVSTDSAGSSYSISGILGITSPSADTNKRKRDEDHLKVSWGPLLPTLAGLPGLHFENFCLPNRFVITPRRRYPIHQAQYSCLGVLPTVCWNGYHKKAVLSPRNSRMVCSPVTVRIAPPDRRFSRSAIPEQIISSTLSSPSSNAPDPCAKETVLSALKEKEKKRTVEEEDQIFLDGQENKRRRHDSSGSGHSAFEPLVANGVPASFVPKPGSLKRGLNSQSSDDHLNKRSRSSSMSSLTGAYASGIPSSSRNAITSSYSSTRGISQLWKRNGPSSSPFSSPASSRSQTPERPAKKIREEELCHHSSSSTPLAADRESQGEKAADTTPRKKQNSNSQSTPGSSGQRKRKVQLLPSRRGEQLTLPPPPQLGYSITAEDLDLEKKASLQWFNQALEDKSDAASNSVTETPPITQPSFTFTLPAAAPASPPTSLLAPSTNPLLESLKKMQTPPSLPPCPESAGAATTEALSPPKTPSLLPPLGLSQSGPPGLLPSPSFDSKPPTTLLGLIPAPSMVPATDTKAPPTLQAETATKPQATSAPSPAPKQSFLFGTQNTSPSSPAAPAASSAPPMFKPIFTAPPKSEKEGPTPPGPSVTATAPSSSSLPTTTSTTAPTFQPVFSSMGPPASVPLPAPFFKQTTTPATAPTTTAPLFTGLASATSAVAPITSASPSTDSASKPAFGFGINSVSSSSVSTTTSTATAASQPFLFGAPQASAASFTPAMGSIFQFGKPPALPTTTTVTTFSQSLHTAVPTATSSSAADFSGFGSTLATSAPATSSQPTLTFSNTSTPTFNIPFGSSAKSPLPSYPGANPQPAFGAAEGQPPGAAKPALAPSFGSSFTFGNSAAPAAAPTPAPPSMIKVVPAYVPTPIHPIFGGATHSAFGLKATASAFGAPASSQPAFGGSTAVFFGAATSSGFGATTQTASSGSSSSVFGSTTPSPFTFGGSAAPAGSGSFGINVATPGSSTTTGAFSFGAGQSGSTATSTPFAGGLGQNALGTTGQSTPFAFNVSSTTESKPVFGGTATPTFGLNTPAPGVGTSGSSLSFGASSAPAQGFVGVAPFGSAALSFSIGAGSKTPGARQRLQARRQHTRKK-

1. Kawamata N, Ogawa S, Zimmermann M, Niebuhr B, Stocking C, Sanada M, Hemminki K, Yamatomo G, Nannya Y, Koehler R, et al: **Cloning of genes involved in chromosomal translocations by high-resolution single nucleotide polymorphism genomic microarray.** *Proc Natl Acad Sci U S A* 2008, **105:**11921-11926.

2. Coyaud E, Struski S, Dastugue N, Brousset P, Broccardo C, Bradtke J: **PAX5-AUTS2 fusion resulting from t(7;9)(q11.2;p13.2) can now be classified as recurrent in B cell acute lymphoblastic leukemia.** *Leuk Res* 2010, **34:**e323-325.

3. Denk D, Nebral K, Bradtke J, Pass G, Moricke A, Attarbaschi A, Strehl S: **PAX5-AUTS2: a recurrent fusion gene in childhood B-cell precursor acute lymphoblastic leukemia.** *Leuk Res* 2012, **36:**e178-181.

4. Bousquet M, Broccardo C, Quelen C, Meggetto F, Kuhlein E, Delsol G, Dastugue N, Brousset P: **A novel PAX5-ELN fusion protein identified in B-cell acute lymphoblastic leukemia acts as a dominant negative on wild-type PAX5.** *Blood* 2007, **109:**3417-3423.

5. Mullighan CG, Zhang J, Harvey RC, Collins-Underwood JR, Schulman BA, Phillips LA, Tasian SK, Loh ML, Su X, Liu W, et al: **JAK mutations in high-risk childhood acute lymphoblastic leukemia.** *PNAS supplements* 2009, **106:**9414-9418.

6. Coyaud E, Struski S, Prade N, Familiades J, Eichner R, Quelen C, Bousquet M, Mugneret F, Talmant P, Pages MP, et al: **Wide diversity of PAX5 alterations in B-ALL: a Groupe Francophone de Cytogenetique Hematologique study.** *Blood* 2010, **115:**3089-3097.

7. Nebral K, Denk D, Attarbaschi A, Konig M, Mann G, Haas OA, Strehl S: **Incidence and diversity of PAX5 fusion genes in childhood acute lymphoblastic leukemia.** *Leukemia* 2009, **23:**134-143.
